# Predictors and Prognostic Factors of Heart Failure with Improved Ejection Fraction

**DOI:** 10.31083/j.rcm2508280

**Published:** 2024-08-08

**Authors:** Nilian Wu, Xueyan Lang, Yanxiu Zhang, Bing Zhao, Yao Zhang

**Affiliations:** ^1^Department of Cardiology, The Second Affiliated Hospital of Harbin Medical University, 150001 Harbin, Heilongjiang, China; ^2^Key Laboratory of Myocardial Ischemia, Ministry of Education, Harbin Medical University, 150001 Harbin, Heilongjiang, China

**Keywords:** heart failure, left ventricular ejection fraction, heart failure with improved ejection fraction, predictor, prognostic factors

## Abstract

**Background:**

Heart failure with reduced ejection fraction (HFrEF) patients who have improved ejection fraction have a better prognosis than those with persistently reduced ejection fraction. This study aimed to analyze the predictors for progression of patients with HFrEF to heart failure with improved ejection fraction (HFimpEF), as well as their characteristics and analyze predictors for prognosis.

**Methods:**

A retrospective analysis was conducted on 1251 patients with HFrEF at baseline, who also had a second echocardiogram ≥3 months. After left ventricular ejection fraction (LVEF) reassessment, patients were separated into the HFimpEF group (n = 408) and the persistent HFrEF group (n = 611). The primary endpoint was a composite of cardiovascular death or heart failure hospitalization.

**Results:**

Multivariate logistic regression showed that without history of alcohol consumption (OR: 0.47, 95% CI: 0.28–0.78), non-New York Heart Association (NYHA) class III–IV (OR: 0.28, 95% CI: 0.15–0.52), without dilated cardiomyopathy (OR: 0.47, 95% CI: 0.26–0.84), concomitant hypertension (OR: 1.53, 95% CI: 1.02–2.29), β-blockers use (OR: 2.29, 95% CI: 1.54–3.43), and lower uric acid (OR: 0.999, 95% CI: 0.997–1.000) could predict LVEF improvement. Kaplan-Meier curves demonstrated that HFimpEF patients had a significantly lower incidence of adverse events than HFrEF patients (log Rank *p* < 0.001). Multivariate Cox regression found that older age (HR: 1.04, 95% CI: 1.02–1.06), NYHA class III–IV (HR: 2.25, 95% CI: 1.28–3.95), concomitant valvular heart disease (HR: 1.98, 95% CI: 1.01–3.85), and higher creatinine (HR: 1.003, 95% CI: 1.001–1.004) were independent risk factors for the primary endpoint in HFimpEF patients.

**Conclusions:**

HFrEF patients without a history of alcohol consumption, non-NYHA class III–IV, without dilated cardiomyopathy, concomitant hypertension, β-blockers use, and lower uric acid were more likely to have LVEF improvement. Although the prognosis of HFimpEF patients was better than that of HFrEF patients, older age, NYHA class III–IV, concomitant valvular heart disease, and higher creatinine were still risk factors for cardiovascular events in HFimpEF patients.

## 1. Introduction

Left ventricular ejection fraction (LVEF) is one of the most important indicators to measure cardiac function of patients with heart failure (HF). It is often used as the critical basis for the classified diagnosis and treatment of patients with HF. Compared to other types of heart failure, heart failure with reduced ejection fraction (HFrEF) often has increased rates of mortality and rehospitalization for heart failure [1]. Patients with HFrEF benefit from treatment and may experience improved ejection fraction in later stages. In 2021, the international heart failure societies jointly issued the Universal Definition and Classification of Heart Failure, proposing heart failure with improved ejection fraction (HFimpEF) [2]. HFimpEF is defined as symptomatic HF with a baseline LVEF ≤40% and >40% on a second LVEF measurement with at least a 10% increase from baseline LVEF [2].

A meta-analysis study found that HFimpEF was associated with a 56% reduction in mortality and a 60% reduction in cardiac hospitalization compared to HFrEF [3]. Despite the varying criteria used to define HFimpEF in this meta-analysis, the potential benefit of LVEF improvement was still recognized. With the advancement of diagnostic and therapeutic technologies, more patients with HFrEF may transition into HFimpEF following treatment. HFimpEF-related clinical studies currently remain few, and there are significant limitations in the perception of patients with HFimpEF. Therefore, this study retrospectively analyzed Chinese patients with HFimpEF based on the latest definition of HFimpEF to provide more reference data for the identification and standardized management of patients with HFimpEF.

## 2. Materials and Methods

### 2.1 Subjects

The investigation conforms with the principles outlined in the Declaration of Helsinki. All patients signed an informed consent form and the study protocol was approved by the HMUSAH Ethics Committee (YJSKY2022-317).

This single-center and retrospective study included patients with HFrEF who were hospitalized at our center between May 2017 and June 2022. Inclusion criteria included: (1) meeting the diagnostic criteria for HFrEF in the 2021 ESC Guidelines for the diagnosis and treatment of acute and chronic heart failure [4]: (a) symptoms and/or signs of HF; (b) N-terminal pro brain natriuretic peptide (NT-proBNP) ≥300 pg/mL; (c) LVEF ≤40%; (2) age ≥18 years; (3) the presence of a second echocardiography ≥3 months from baseline, except for baseline echocardiography. Exclusion criteria included: (1) changes in LVEF classifications beyond the diagnostic criteria for HFrEF and HFimpEF; (2) isolated right HF, congenital heart disease, perinatal cardiomyopathy, HF due to Keshan disease; (3) history of heart transplantation or left ventricular assist device implantation; (4) lost to follow-up during the study period.

**Fig. 1.**
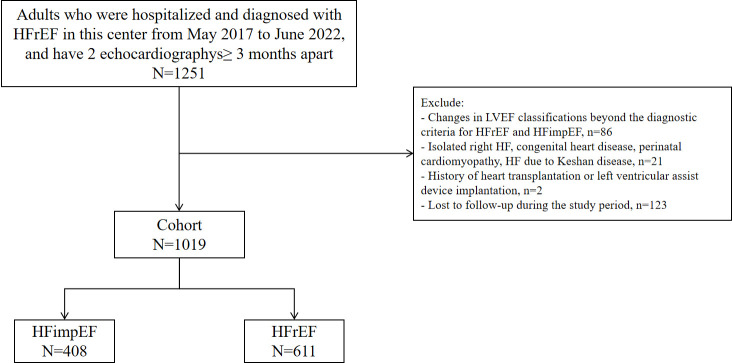
**Flow chart of study participant selection**. HFrEF, heart failure with reduced ejection fraction; LVEF, left ventricular ejection fraction; HF, heart failure; HFimpEF, heart failure with improved ejection fraction.

This study initially included HFrEF patients who satisfied the inclusion criteria, and 1019 patients remained for analysis after the exclusion criteria (Fig. 1).

### 2.2 Research Method

(1) Baseline data collection: General characteristics, comorbidities, laboratory test results, diagnostic findings, treatment modalities, and other relevant data of the patients were acquired from the hospital medical records system. (2) Grouping: LVEF was obtained from the echocardiographic findings of patients. If there were multiple echocardiogram results, the most recent result from the baseline was used for grouping. Baseline LVEF ≤40%, and a second LVEF measurement ≤40% were the inclusion criteria for the HFrEF group, while baseline LVEF ≤40% and a second LVEF measurement >40% with absolute improvement from baseline ≥10% were the inclusion criteria for the HFimpEF group.

### 2.3 Follow-up Method and Endpoint Events

The follow-up period began after each patient completed a second echocardiogram. Follow-up was conducted through face-to-face interviews and/or phone interviews. Follow-up intervals were 1 week, 1 month, 3 months, 1 year, and annually thereafter until March 2023. The primary endpoint was a composite of cardiovascular death or HF hospitalization, and the secondary endpoint was all-cause mortality.

### 2.4 Data Analysis

Continuous variables were expressed as mean and standard deviation or median [interquartile range, IQR], and inter-group comparisons used Student’s *t*-test or Mann-Whitney U test. Categorical variables were presented as frequencies and percentages (%), and inter-group comparisons used the Chi-Square test. Survival curves were plotted by the Kaplan-Meier method, and the log-rank test was used for differences in survival rates between the groups. Predictors of LVEF improvement were determined using logistic regression analysis. The Cox proportional hazards model was utilized to assess prognostic factors in the HFimpEF and HFrEF groups. The following variables were studied: sex, age, smoking history, alcohol consumption history, New York Heart Association (NYHA) class III–IV, comorbidities (coronary heart disease, dilated cardiomyopathy, valvular heart disease, atrial fibrillation, hypertension, diabetes), treatment modalities (angiotensin receptor neprilysin inhibitor (ARNI)/angiotensin converting enzyme inhibitor (ACEI)/angiotensin receptor blocker (ARB), β-blockers, aldosterone receptor antagonists), and laboratory results (uric acid, creatinine, potassium, sodium, chloride, hemoglobin, platelet distribution width, plateletcrit, red blood cell distribution width/albumin ratio (RAR)). These variables were included in the univariate analysis to assess predictors for improvement of ejection fraction and prognosis, respectively. The multivariate analysis further included variables with a *p*-value less than 0.05 from the results of the univariate analysis. For evaluating predictors of ejection fraction improvement, the potential influence of the time interval between the second echocardiogram and baseline echocardiogram was taken into consideration, and the echocardiogram time intervals were additionally included in the multivariate analysis. SPSS software (Version 25; IBM, Armonk, NY, USA) was employed to complete the statistical analysis. A *p*-value < 0.05 was considered statistically significant.

## 3. Results

### 3.1 Baseline Characteristics

Among the 1019 patients with HFrEF at baseline, patients were divided into the HFimpEF group (408 patients, 40%) and the HFrEF group (611 patients, 60%) based on the ejection fraction on the second echocardiography. Table 1 shows the comparisons of baseline characteristics between the HFimpEF group and the HFrEF group. Compared with the HFrEF group, patients with HFimpEF had a higher incidence of comorbidities, such as coronary heart disease, hypertension, and diabetes, and were more likely to receive percutaneous coronary intervention (PCI)/coronary artery bypass grafting (CABG), and β-blockers treatment. Additionally, the HFimpEF group had higher body mass index (BMI) and plateletcrit. Patients with HFrEF were more prone to have a history of alcohol consumption; they were also more likely to be in NYHA class III–IV and had a greater incidence of dilated cardiomyopathy. Additionally, they were more likely to receive inotropes, and had higher NT-proBNP, uric acid, creatinine, potassium, and platelet distribution width values than patients with HFimpEF.

**Table 1.** **Baseline characteristics of patients in HFimpEF group compared with HFrEF group**.

Characteristics		HFimpEF	HFrEF	*p* value
		(n = 408)	(n = 611)	
Age (years)*		62 (53, 69)	62 (54, 69)	0.885
Sex (male) (%)*		290 (71.08)	436 (71.36)	0.923
Smoking history (%)*		151 (37.01)	222 (36.33)	0.826
Alcohol consumption history (%)*		56 (13.73)	126 (20.62)	0.005
BMI (kg/$m^{2}$)		25.56 ± 4.91	24.43 ± 5.08	0.003
Edema (%)		139 (34.07)	230 (37.64)	0.245
NYHA class III–IV (%)*		299 (73.28)	528 (86.42)	<0.001
Comorbidities				
	Coronary heart disease (%)*	275 (67.40)	351 (57.45)	0.001
	Dilated cardiomyopathy (%)*	52 (12.75)	146 (23.90)	<0.001
	Valvular heart disease (%)*	24 (5.88)	28 (4.58)	0.356
	Atrial fibrillation (%)*	88 (21.57)	115 (18.82)	0.282
	Hypertension (%)*	199 (48.78)	220 (36.01)	<0.001
	Diabetes (%)*	128 (31.37)	140 (22.91)	0.003
Treatment				
	PCI/CABG (%)	155 (37.99)	77 (12.60)	<0.001
	ICD/CRT (%)	12 (2.94)	19 (3.12)	0.874
	ARNI/ACEI/ARB (%)*	281 (68.87)	429 (70.33)	0.620
	β-blockers (%)*	290 (71.08)	377 (61.70)	0.002
	Aldosterone receptor antagonists (%)*	311 (76.23)	478 (78.23)	0.453
	SGLT2i (%)	12 (2.94)	13 (2.13)	0.411
	Diuretics (%)	335 (82.11)	524 (85.76)	0.116
	Inotropes (%)	277 (67.89)	467 (76.43)	0.003
Laboratory				
	NT-proBNP (pg/mL)	3989 (1748, 7936)	4604.50 (2410.50, 9862.20)	0.006
	Uric acid (µmol/L)*	395.50 (311.33, 501.95)	444.70 (341.40, 554)	<0.001
	Creatinine (µmol/L)*	91 (76, 109)	95 (79, 118)	0.014
	Potassium (mmol/L)*	3.90 (3.60, 4.30)	4 (3.70, 4.40)	0.016
	Sodium (mmol/L)*	139.85 (136.93, 142)	139.80 (136.90, 142)	0.689
	Chloride (mmol/L)*	103 (101, 106)	103 (101, 105)	0.747
	Hemoglobin (g/L)*	140 (126, 153)	140 (127, 152)	0.881
	PDW (%)*	14.20 (12.68, 15.90)	14.50 (13.10, 16.05)	0.023
	PCT (%)*	0.25 (0.21, 0.30)	0.24 (0.20, 0.28)	0.001
	RAR*	3.38 (3.10, 3.72)	3.41 (3.13, 3.81)	0.479

* Variables marked with an asterisk were included in univariate logistic regression and Cox regression analysis. Abbreviations: HFimpEF, heart failure with improved ejection fraction; HFrEF, heart failure with reduced ejection fraction; BMI, body mass index; NYHA, New York Heart Association; PCI, percutaneous coronary intervention; CABG, coronary artery bypass grafting; ICD, implantable cardioverter defibrillator; CRT, cardiac resynchronization therapy; ACEI, angiotensin converting enzyme inhibitor; ARB, angiotensin receptor blocker; ARNI, angiotensin receptor neprilysin inhibitor; SGLT2i, sodium-glucose transporter 2 inhibitor; NT-proBNP, N-terminal pro brain natriuretic peptide; PDW, platelet distribution width; PCT, plateletocrit; RAR, red blood cell distribution width/albumin ratio; n, number.

### 3.2 Predictors of Ejection Fraction Improvement

The median time interval between the two echocardiograms was 8.17 (7.53, 8.63) months. Among the 1019 patients with HFrEF, 408 patients (40%) had a ≥10% improvement in LVEF from baseline, which conforms with the definition of HFimpEF used in this study. Univariate logistic regression showed that without history of alcohol consumption, non-NYHA class III–IV, concomitant coronary heart disease, without dilated cardiomyopathy, concomitant hypertension, concomitant diabetes, use of β-blockers, lower uric acid, lower platelet distribution width, and higher plateletcrit were predictors of improved ejection fraction (*p* < 0.05). After including echocardiogram interval, age, sex, and the above variables, the adjusted multivariate logistic regression results showed that without history of alcohol consumption, non-NYHA class III–IV, without dilated cardiomyopathy, concomitant hypertension, use of β-blockers, and lower uric acid remained predictors of improved ejection fraction (*p* < 0.05). The results are presented in Table 2.

**Table 2.** **Logistic regression of baseline characteristics associated with LVEF improvement**.

	Univariate analysis		Multivariate analysis	
	OR (95% CI)	*p* value	OR (95% CI)	*p* value
Age	1.00 (0.99–1.01)	0.329	0.99 (0.97–1.01)	0.283
Sex (male)	0.99 (0.75–1.30)	0.923	1.08 (0.66–1.76)	0.773
Ultrasound interval	0.97 (0.96–0.99)	<0.001	0.95 (0.93–0.96)	<0.001
Alcohol consumption history	0.61 (0.43–0.86)	0.005	0.47 (0.28–0.78)	0.004
NYHA class III–IV	0.43 (0.31–0.59)	<0.001	0.28 (0.15–0.52)	<0.001
Coronary heart disease	1.53 (1.18–1.99)	0.001	0.98 (0.58–1.63)	0.938
Dilated cardiomyopathy	0.47 (0.33–0.65)	<0.001	0.47 (0.26–0.84)	0.012
Hypertension	1.69 (1.31–2.19)	<0.001	1.53 (1.02–2.29)	0.040
Diabetes	1.54 (1.16–2.04)	0.003	0.94 (0.60–1.48)	0.794
β-blockers	1.53 (1.17–2.00)	0.002	2.29 (1.54–3.43)	<0.001
Uric acid	0.998 (0.997–0.999)	<0.001	0.999 (0.997–1.000)	0.046
PDW	0.94 (0.89–0.99)	0.026	1.07 (0.98–1.16)	0.141
PCT	16.64 (2.92–98.13)	0.002	20.02 (0.89–510.93)	0.065

Abbreviations: NYHA, New York Heart Association; PDW, platelet distribution width; PCT, plateletocrit; LVEF, left ventricular ejection fraction; OR, odds ratio; CI, confidence interval.

### 3.3 Survival Comparison

**Fig. 2.**
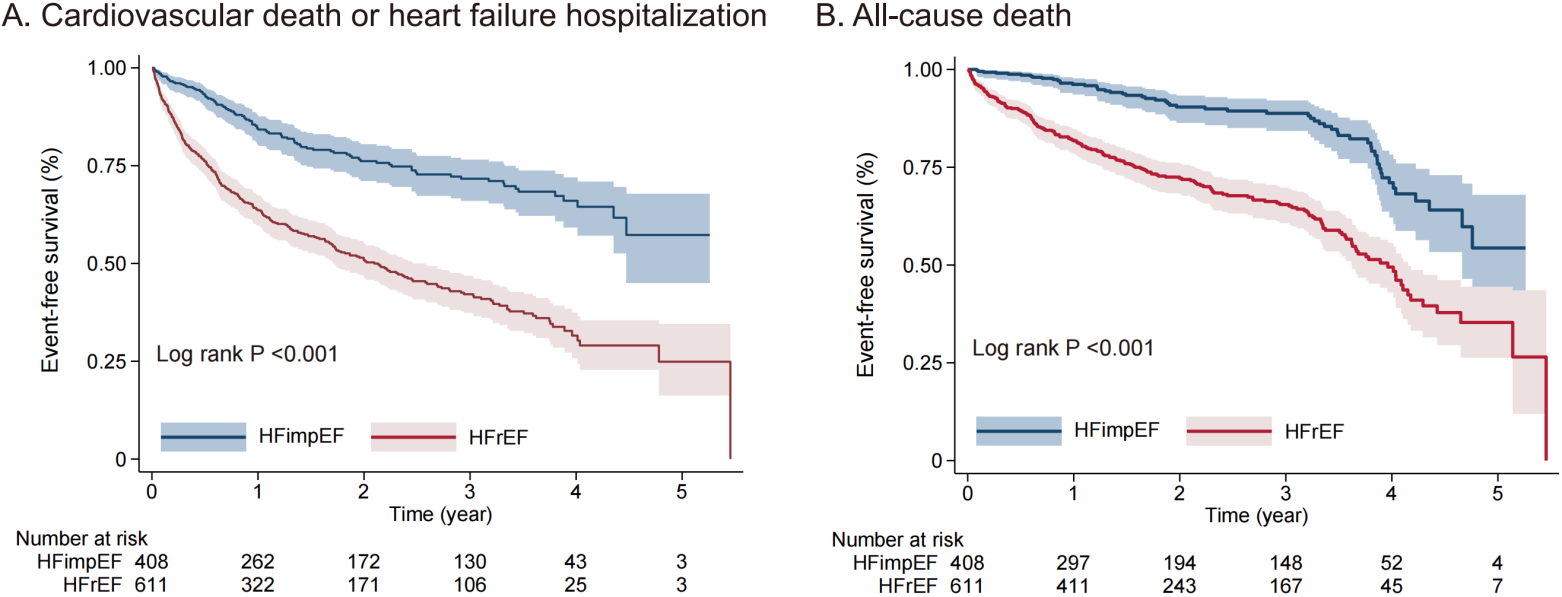
**Kaplan-Meier curve Showing the composite of cardiovascular death or heart failure hospitalization (A) and all-cause death (B)**. HFimpEF, heart failure with improved ejection fraction; HFrEF, heart failure with reduced ejection fraction.

During the follow-up period of 2.18 (2.13, 2.46) years following the second echocardiogram, there were a total of 100 cases of cardiovascular death or HF hospitalization, along with 57 cases of all-cause mortality in patients with HFimpEF. Three hundred eighteen patients in the HFrEF group developed cardiovascular death or HF hospitalization, and 210 patients died of all causes. Kaplan-Meier curves revealed a significant difference in survival time between the two groups for both the primary and secondary endpoints, with HFimpEF patients having a significantly more favorable prognosis than patients with HFrEF (*p* < 0.001). The results are shown in Fig. 2A,B.

### 3.4 Prognostic Factors in Patients with HFimpEF

#### 3.4.1 Primary Endpoint

The results of univariate Cox regression revealed that older age, NYHA class III–IV, concomitant valvular heart disease, higher creatinine levels, higher potassium levels, and lower plateletcrit were risk factors for cardiac vascular death or HF hospitalization in patients with HFimpEF (*p* < 0.05). Multivariate Cox regression analysis, including sex and above factors, revealed that older age, NYHA class III–IV, concomitant valvular heart disease, and higher creatinine remained independent risk factors for cardiovascular death or HF hospitalization in patients with HFimpEF (*p <* 0.05).The results are presented in Table 3.

**Table 3.** **Hazard ratios (95% CIs) of primary endpoint and secondary endpoint with HFimpEF**.

		Univariate analysis		Multivariate analysis	
		HR (95% CI)	*p* value	HR (95% CI)	*p* value
Cardiovascular death or heart failure hospitalization					
	Age	1.04 (1.02–1.06)	<0.001	1.04 (1.02–1.06)	<0.001
	Sex (male)	0.99 (0.64–1.52)	0.946	1.15 (0.71–1.84)	0.578
	NYHA class III–IV	2.38 (1.39–4.07)	0.002	2.25 (1.28–3.95)	0.005
	Valvular heart disease	2.10 (1.09–4.04)	0.026	1.98 (1.01–3.85)	0.046
	Creatinine	1.003 (1.001–1.004)	0.009	1.003 (1.001–1.004)	0.009
	Potassium	1.46 (1.12–1.89)	0.004	1.26 (0.94–1.68)	0.125
	PCT	0.04 (0.00–0.92)	0.044	0.23 (0.01–5.53)	0.368
All-cause mortality					
	Age	1.04 (1.02–1.07)	<0.001	1.04 (1.02–1.07)	0.002
	Sex (male)	1.02 (0.57–1.82)	0.952	1.35 (0.72–2.51)	0.346
	NYHA class III–IV	2.33 (1.14–4.75)	0.020	2.02 (0.92–4.42)	0.079
	Valvular heart disease	3.25 (1.53–6.90)	0.002	3.36 (1.49–7.61)	0.004
	Atrial fibrillation	2.11 (1.21–3.68)	0.008	1.32 (0.73–2.39)	0.358
	β-blockers	0.52 (0.31–0.89)	0.016	0.58 (0.33–0.99)	0.047
	Creatinine	1.002 (1.000–1.005)	0.043	1.002 (1.000–1.005)	0.110
	PDW	1.15 (1.04–1.27)	0.005	1.12 (1.01–1.24)	0.028
	RAR	1.58 (1.15–2.16)	0.005	1.18 (0.77–1.80)	0.440

Abbreviations: NYHA, New York Heart Association; PCT, plateletocri; PDW, platelet distribution widtht; RAR, red blood cell distribution width/albumin ratio; HFimpEF, heart failure with improved ejection fraction; HR, hazard ratio; CI, confidence interval.

#### 3.4.2 Secondary Endpoint

Univariate Cox regression analysis revealed that older age, NYHA class III–IV, concomitant valvular heart disease, concomitant atrial fibrillation, non-use of β-blockers, higher creatinine levels, higher platelet distribution width, and higher RAR were risk factors for all-cause mortality in patients with HFimpEF (*p* < 0.05). Multivariate Cox regression analysis, including sex and the above factors, revealed that older age, concomitant valvular heart disease, non-use of β-blockers, and higher platelet distribution width remained independent risk factors for all-cause mortality in patients with HFimpEF (*p* < 0.05). The results are presented in Table 3.

### 3.5 Prognostic Factors in Patients with HFrEF

#### 3.5.1 Primary Endpoint

Univariate Cox regression analysis found that older age, higher creatinine levels, and lower chloride levels were risk factors for cardiovascular death or HF hospitalization in patients with HFrEF (*p* < 0.05). Multivariate Cox regression analysis, including sex and above factors, revealed that older age, higher creatinine levels, and lower chloride levels remained independent risk factors for cardiovascular death or HF hospitalization in patients with HFrEF (*p* < 0.05). The results are presented in Table 4.

**Table 4.** **Hazard ratios (95% CIs) of primary endpoint and secondary endpoint with HFrEF**.

		Univariate analysis		Multivariate analysis	
		HR (95% CI)	*p* value	HR (95% CI)	*p* value
Cardiovascular death or heart failure hospitalization					
	Age	1.01 (1.01–1.02)	0.004	1.01 (1.01–1.02)	0.003
	Sex (male)	1.24 (0.97–1.60)	0.092	1.23 (0.95–1.60)	0.109
	Creatinine	1.002 (1.001–1.004)	<0.001	1.002 (1.001–1.004)	0.003
	Chloride	0.97 (0.94–0.99)	0.008	0.97 (0.95–1.00)	0.022
All-cause mortality					
	Age	1.03 (1.02–1.04)	<0.001	1.03 (1.01–1.04)	<0.001
	Sex (male)	1.20 (0.88–1.65)	0.245	1.27 (0.90–1.80)	0.178
	Smoking history	0.74 (0.56–1.00)	0.047	0.76 (0.56–1.02)	0.067
	Creatinine	1.003 (1.002–1.005)	<0.001	1.003 (1.001–1.005)	0.002
	Potassium	1.39 (1.11–1.74)	0.005	1.20 (0.95–1.52)	0.123
	Sodium	0.96 (0.94–0.99)	0.009	1.00 (0.96–1.04)	0.957
	Chloride	0.96 (0.94–1.00)	0.022	0.97 (0.93–1.02)	0.203
	Hemoglobin	0.99 (0.99–1.00)	0.046	1.00 (0.99–1.01)	0.531
	RAR	1.47 (1.23–1.75)	<0.001	1.33 (1.08–1.65)	0.007

Abbreviations: RAR, red blood cell distribution width/albumin ratio; HFrEF, heart failure with reduced ejection fraction; HR, hazard ratio; CI, confidence interval.

#### 3.5.2 Secondary Endpoint

Univariate Cox regression analysis revealed that older age, without smoking history, higher creatinine levels, higher potassium levels, lower sodium levels, lower chloride levels, lower hemoglobin levels, and higher RAR were risk factors for all-cause mortality in patients with HFrEF (*p* < 0.05). Multivariate Cox regression analysis, including sex and above factors, revealed that older age, higher creatinine levels, and higher RAR remained independent risk factors for all-cause mortality in patients with HFrEF (*p* < 0.05). The results are presented in Table 4.

## 4. Discussion

Our study was based on the latest definition of heart failure with improved ejection fraction, demonstrating the possibility of HFrEF progressing to HFimpEF and having a better prognosis. We found that HFrEF patients without a history of alcohol consumption, non-NYHA class III–IV, without dilated cardiomyopathy, concomitant hypertension, β-blockers use, and lower uric acid were more likely to have ejection fraction improvement. In addition, we identified older age, NYHA class III–IV, concomitant valvular heart disease, and higher creatinine as risk factors for future cardiovascular events in HFimpEF patients.

This study found that 40% of HFrEF patients had ≥10% improvement in LVEF from baseline on the second echocardiogram after receiving treatment. A previous study including 3124 patients with HFrEF showed that 1174 (37.6%) patients with HFrEF exhibited LVEF recovery ≥10% [5]. Because there is no uniform definition of HFimpEF among studies [3, 5, 6, 7], inclusion and exclusion criteria are not consistent, making it challenging to estimate the true incidence of HFimpEF. The baseline analysis of this study revealed no significant differences in demographic characteristics between the two groups, and little difference existed in the usage of drugs recommended by most guidelines, except for β-blockers and cardiotonic agents. Patients with persistent HFrEF are more prone to dilated cardiomyopathy, while patients with HFimpEF are more prone to coronary heart disease, hypertension, and diabetes.

Based on our study, we found that neither group of patients reached 80% in the use of ARNI/ACEI/ARB, β-blockers, and aldosterone receptor antagonists. Similar findings have also been observed in real-world studies, where HFrEF patients did not perform well in terms of following guideline-recommended standard drug therapy [8, 9, 10]. This may be because real-world medication use in heart failure patients is often limited by personalized medication, such as concerns about drug-related adverse reactions such as renal function deterioration, hyperkalemia, and hypotension, which may make it difficult to use or require delayed initiation; in addition, physicians may be more concerned with alleviating current symptoms in frail elderly heart failure patients than improving the long-term prognosis [11]. The need for regular review and long-term follow-up of patients with heart failure to complete guideline-directed standard drug therapy should be emphasized as much as possible.

The adjusted multivariate logistic regression in this study revealed that ejection fraction was more prone to be improved in patients with hypertension and β-blockers, while ejection fraction was more likely to be consistently decreased in patients with alcohol consumption history, NYHA class III–IV, concomitant dilated cardiomyopathy, and higher uric acid levels. The decrease in ejection fraction in patients with HF is often related to persistent adverse myocardial remodeling, which is affected by various factors, including hemodynamic changes and neurohumoral activation. After treatment, partial recovery of myocardial structure and function may occur in patients with HF, but the specific mechanism of partial reverse remodeling of this myocardium is not fully understood [12]. Previous studies have retrospectively evaluated left ventricular systolic function recovery in 98 patients with idiopathic cardiomyopathy, with 19 patients (19%) showing improvement during follow-up and hypertension history as an independent predictor of improvement [13]. This is similar to our study, where we believe that the standardized management of hypertension is beneficial for improving ejection fraction. The use of β-blockers contributes to the transition from HFrEF patients to HFimpEF patients. It has also previously been shown that LVEF increases from 33 ± 8% to 54 ± 6% in patients with left ventricular systolic dysfunction after using β-blockers [14]. β-blockers may improve ejection fraction in patients with chronic HF because of their heart rate-lowering effect [15]. Also, this improvement in ejection fraction may be closely associated with alterations in myocardial collagen metabolism and, to some extent, independent of a decrease in heart rate [16]. A history of alcohol consumption can also influence the improvement of ejection fraction. Impaired left ventricular function has been observed in individuals who drink alcohol in previous studies. Animal experiments also suggest that alcohol may induce myocardial atrophy through pro-inflammatory and profibrotic mechanisms [17, 18]. In patients with HFrEF, NYHA class III–IV patients showed a higher mortality risk than class II patients [19]. Our results suggest that NYHA class III–IV may also indicate a poor likelihood of ejection fraction improvement in such patients with HFrEF. Dilated cardiomyopathy is a structural cardiomyopathy characterized by thinning and dilating the ventricular wall with a wide range of underlying etiologies and challenges associated with early intervention [20]. Additionally, the structural features of dilated cardiomyopathy also include mitral regurgitation, and asynchronous ventricular contraction, which can lead to the persistence of left ventricular remodeling [21]. Therefore, patients with dilated cardiomyopathy are more likely to have persistently reduced ejection fraction. This study also revealed that patients with higher uric acid levels were more prone to have persistent reductions in ejection fraction. Previous studies found that hyperuricemia might reflect hyperactive oxidative stress in HF [22].

This study found that patients with HFimpEF showed statistically significantly lower cardiovascular death or HF hospitalization rates than patients with HFrEF, as was all-cause mortality. Although patients with HFimpEF have a favorable prognosis, it is important to identify those risk factors which result in adverse outcomes in patients with HFimpEF. The multivariate Cox regression analysis revealed that older age and valvular heart disease independently predicted cardiovascular death, HF hospitalization, and all-cause mortality in patients with HFimpEF. Moreover, NYHA class III–IV and higher creatinine levels were independent risk factors for cardiovascular death or HF hospitalization; the absence of β-blockers and higher platelet distribution width levels were independent risk factors for all-cause mortality. Concomitant valvular heart disease also complicates HF since valvular heart disease may cause chronic hemodynamic abnormalities, leading to ventricular wall responses to pressure overload [23, 24]. Although some patients with baseline NYHA class III–IV also regained ejection fraction above 40% after treatment, the findings suggested that these HFimpEF patients were prone to cardiovascular events. Previous studies reported that using β-blockers may prevent the occurrence of left ventricular systolic function dysfunction in patients with dilated cardiomyopathy and recovered ejection fraction [25]. Our results also support that β-blockers are essential for ejection fraction improvement and prognosis in HFimpEF patients. Platelet distribution width is a simple parameter in routine blood tests and can be used as a biomarker of platelet activation. Our findings were in accordance with previous studies showing that high levels of platelet distribution width may predict an unfavorable prognosis in HF patients [26]. Heart failure patients should be monitored for signs of platelet activation, which can may reflect lower liver and kidney blood flow perfusion, increased sympathetic nervous system excitation, and impaired endothelial function [27]. In addition, higher platelet levels indicate a higher potential for thrombosis in patients, which may lead to increased myocardial microvascular circulation injury [28].

This study also analyzed risk factors for an unfavorable prognosis in patients with persistent HFrEF and found that older age and higher creatinine levels independently predicted cardiovascular death or HF hospitalization and all-cause mortality in patients with HFrEF. Furthermore, lower chloride was an independent risk factor for cardiovascular death or HF hospitalization; and higher RAR was an independent risk factor for all-cause mortality. Electrolyte imbalance often occurs in HF patients, and serum chloride levels have recently received attention in HF-related studies. Abnormal chloride levels may facilitate HF progression through mechanisms such as neurohormonal activation and diuretic resistance [29]. Low serum chloride is closely and independently related to increased mortality, all-cause mortality, and HF hospitalization [30]. Chronic HF patients also experience long-term, low-grade circulating inflammation in addition to electrolyte abnormalities. The levels of inflammatory factors may reflect the degree of myocardial injury [31], making it critical to identify appropriate inflammatory biomarkers. RAR is a novel inflammatory biomarker that correlates positively with NT-proBNP levels, showing its ability to predict short-term and long-term mortality in HF patients [32]. This study found that RAR might also predict all-cause mortality in patients with persistent HFrEF but cannot predict adverse events in patients with HFimpEF. We conclude that patients with HFimpEF are less likely to be in a positive feedback loop of inflammation and HF exacerbation for longer periods, and thus transient baseline inflammation levels have limited predictive significance for the prognosis of patients with HFimpEF.

These findings indicate that older age and higher creatinine levels were common prognostic factors in patients with HFimpEF and persistent HFrEF. Regardless of the type of HF, the treatment of elderly HF patients has been a challenging issue. Cardiac vascular stiffness increases with aging, and both responsiveness to adrenergic stimulation and left ventricular diastolic filling are decreased [33]. Moreover, older age may be associated with more chronic diseases, and other systemic diseases can interact with HF or even promote deterioration. Changes in renal hemodynamics can affect the balance of the neuroendocrine system, resulting in further increases in profibrotic neurohormones and aggravated ventricular remodeling in HF patients [34]. Previous studies reported that every 0.5 mg/dL increase in creatinine increased mortality by 15% in HF patients [35]. The current study also revealed that renal dysfunction might be one of the critical risk factors for adverse events in patients with HFimpEF and HFrEF.

Heart failure, as a high-burden chronic disease that requires long-term standardized management, and requires further research. The emergence of HFimpEF demonstrates the potential for patients with HFrEF to progress toward a more favorable outcome. Currently, not enough attention has been paid to HFimpEF, and our study attempts to provide some evidence for the identification and management of patients with HFimpEF. In the future, more prospective, multicenter studies are needed based on a wider patient population to provide comprehensive and reliable evidence-based medicine evidence on HFimpEF.

This study had certain limitations. First, this is a single-center study, and the included patient population is mainly concentrated in Northeast China, which may limit its representativeness. Second, we failed to evaluate the possible benefit of sodium-glucose transporter 2 (SGLT2) inhibitors in patients with HFimpEF or HFrEF, as SGLT2 inhibitors have only been officially approved in China since February 2021 for treating patients with HFrEF. Finally, we did not evaluate other echocardiographic indicators besides LVEF, and failed to follow up on the long-term dynamic changes of LVEF.

## 5. Conclusions

This study showed that HFrEF patients without a history of alcohol consumption, non-NYHA class III–IV, without dilated cardiomyopathy, concomitant hypertension, β-blockers use, and lower uric acid were more prone to have ejection fraction improvement. Patients with HFimpEF had fewer adverse cardiovascular events than patients with HFrEF. Older age and higher creatinine levels were independent risk factors for the primary endpoint in the two groups. Additionally, NYHA class III–IV and concomitant valvular heart disease were independent risk factors for the primary endpoint in patients with HFimpEF; lower chloride was also an independent risk factor for the primary endpoint in patients with HFrEF. In clinical practice, more attention should be paid to the influencing factors that may promote the transition to patients with HFimpEF, and the risk factors that result in adverse events in patients with HFimpEF.

## Data Availability

All data generated or analysed during this study are included in this published article and its supplementary information files.

## Supplementary Material

Supplementary material associated with this article can be found, in the online version, at https://doi.org/10.31083/j.rcm2508280.
