# Supplementary material for: Predictors and Prognostic Factors of Heart Failure with Improved Ejection Fraction
Source: Rev Cardiovasc Med. 2024 Aug 8;25(8):280. doi: 10.31083/j.rcm2508280 (PMC11367010; doi:10.31083/j.rcm2508280)
Supplement: Supplementary file 1 [file 2153-8174-25-8-280-s1.docx]

**Supplementary table 1.Logistic regression of baseline characteristics associated with LVEF** **improvement**

|  | **Univariate analysis** | | **Multivariate analysis** | |
| --- | --- | --- | --- | --- |
|  | **OR(95% CI)** | **P value** | **OR(95%CI)** | **P value** |
| Age | 1.00(0.99-1.01) | 0.329 | 0.99(0.97-1.01) | 0.283 |
| Sex(male) | 0.99(0.75-1.30) | 0.923 | 1.08(0.66-1.76) | 0.773 |
| Ultrasound interval | 0.97(0.96-0.99) | <0.001 | 0.95(0.93-0.96) | <0.001 |
| Smoking history | 1.03(0.79-1.34) | 0.826 |  |  |
| Alcohol consumption history | 0.61(0.43-0.86) | 0.005 | 0.47(0.28-0.78) | 0.004 |
| NYHA class Ⅲ-Ⅳ | 0.43(0.31-0.59) | <0.001 | 0.28(0.15-0.52) | <0.001 |
| Coronary heart disease | 1.53(1.18-1.99) | 0.001 | 0.98(0.58-1.63) | 0.938 |
| Dilated cardiomyopathy | 0.47(0.33-0.65) | <0.001 | 0.47(0.26-0.84) | 0.012 |
| Valvular heart disease | 1.30(0.74-2.28) | 0.357 |  |  |
| Atrial fibrillation | 1.19(0.87-1.62) | 0.282 |  |  |
| Hypertension | 1.69(1.31-2.19) | <0.001 | 1.53(1.02-2.29) | 0.040 |
| Diabetes | 1.54(1.16-2.04) | 0.003 | 0.94(0.60-1.48) | 0.794 |
| ARNI/ACEI/ARB | 0.93(0.71-1.23) | 0.620 |  |  |
| β-blockers | 1.53(1.17-2.00) | 0.002 | 2.29(1.54-3.43) | <0.001 |
| Aldosterone receptor antagonists | 0.89(0.66-1.20) | 0.453 |  |  |
| Uric acid | 0.998(0.997-0.999) | <0.001 | 0.999(0.997-1.000) | 0.046 |
| Creatinine | 1.000(0.999-1.003) | 0.576 |  |  |
| Potassium | 1.20(0.97-1.48) | 0.088 |  |  |
| Sodium | 1.00(0.97-1.03) | 0.947 |  |  |
| Chloride | 0.98(0.95-1.01) | 0.206 |  |  |
| Hemoglobin | 1.000(0.995-1.007) | 0.806 |  |  |
| PDW | 0.94(0.89-0.99) | 0.026 | 1.07(0.98-1.16) | 0.141 |
| PCT | 16.64(2.92-98.13) | 0.002 | 20.02(0.89-510.93) | 0.065 |
| RAR | 0.91(0.77-1.08) | 0.281 |  |  |

**Abbreviations:** NYHA, New York Heart Association; ACEI, angiotensin converting enzyme inhibitor; ARB, angiotensin receptor blocker; ARNI, angiotensin receptor neprilysin inhibitor; PDW, platelet distribution width; PCT, plateletocrit; RAR, red blood cell distribution width/albumin ratio; LVEF, left ventricular ejection fraction; OR, odd ratio; CI, confidence interval.

**Supplementary table 2.Hazard ratios (95% CIs) of primary endpoint and secondary endpoint with HFimpEF and HFrEF**

|  | **HFimpEF** | | | | **HFrEF** | | | | |  |
| --- | --- | --- | --- | --- | --- | --- | --- | --- | --- | --- |
|  | **Univariateanalysis** | | **Multivariateanalysis** | | **Univariateanalysis** | | **Multivariateanalysis** | | |  |
|  | **HR(95%CI)** | **P value** | **HR(95%CI)** | **P value** | **HR(95%CI)** | **P value** | **HR(95%CI)** | | **P value** |  |
| **Cardiovascular death or heart failure hospitalization** | | | | |  |  |  | |  |  |
| Age | 1.04(1.02-1.06) | <0.001 | 1.04(1.02-1.06) | <0.001 | 1.01(1.01-1.02) | 0.004 | 1.01(1.01-1.02) | | 0.003 |  |
| Sex(male) | 0.99(0.64-1.52) | 0.946 | 1.15(0.71-1.84) | 0.578 | 1.24(0.97-1.60) | 0.092 | 1.23(0.95-1.60) | | 0.109 |  |
| Smoking history | 0.78(0.52-1.19) | 0.252 |  |  | 0.82(0.65-1.30) | 0.103 |  | |  |  |
| Alcohol consumption history | 0.75(0.41-1.37) | 0.347 |  |  | 0.99(0.76-1.30) | 0.951 |  | |  |  |
| NYHA class Ⅲ-Ⅳ | 2.38(1.39-4.07) | 0.002 | 2.25(1.28-3.95) | 0.005 | 1.22(0.88-1.71) | 0.238 |  | |  |  |
| Coronary heart disease | 1.06(0.68-1.64) | 0.793 |  |  | 1.02(0.81-1.27) | 0.891 |  | |  |  |
| Dilated cardiomyopathy | 0.56(0.29-1.23) | 0.159 |  |  | 0.94(0.73-1.22) | 0.654 |  | |  |  |
| Valvular heart disease | 2.10(1.09-4.04) | 0.026 | 1.98(1.01-3.85) | 0.046 | 1.22(0.75-1.98) | 0.434 |  | |  |  |
| Atrial fibrillation | 1.47(0.95-2.27) | 0.087 |  |  | 1.18(0.90-1.56) | 0.240 |  | |  |  |
| Hypertension | 1.25(0.84-1.85) | 0.275 |  |  | 1.01(0.80-1.27) | 0.935 |  | |  |  |
| Diabetes | 1.08(0.72-1.64) | 0.707 |  |  | 1.28(0.99-1.64) | 0.056 |  | |  |  |
| ARNI/ACEI/ARB | 0.86(0.57-1.29) | 0.462 |  |  | 0.83(0.66-1.06) | 0.130 |  | |  |  |
| β-blockers | 0.76(0.50-1.15) | 0.196 |  |  | 0.84(0.67-1.04) | 0.114 |  | |  |  |
| Aldosterone receptor antagonists | 1.23(0.76-2.01) | 0.402 |  |  | 0.89(0.68-1.15) | 0.366 |  | |  |  |
| Uric acid | 1.001(1.000-1.002) | 0.194 |  |  | 1.00(1.00-1.00) | 0.294 |  | |  |  |
| Creatinine | 1.003 (1.001 - 1.004) | 0.009 | 1.003(1.001-1.004) | 0.009 | 1.002(1.001-1.004) | < 0.001 | 1.002(1.001-1.004) | | 0.003 |  |
| Potassium | 1.46(1.12-1.89) | 0.004 | 1.26(0.94-1.68) | 0.125 | 1.02(0.86-1.23) | 0.794 |  | |  |  |
| Sodium | 1.00(0.95-1.05) | 0.986 |  |  | 0.98(0.96-1.00) | 0.098 |  | |  |  |
| Chloride | 1.00(0.94-1.05) | 0.842 |  |  | 0.97(0.94-0.99) | 0.008 | 0.97(0.95-1.00) | | 0.022 |  |
| Hemoglobin | 1.00(0.99-1.01) | 0.324 |  |  | 1.00(0.99-1.01) | 0.944 |  | |  |  |
| PDW | 1.06(0.98-1.14) | 0.162 |  |  | 1.04(0.99-1.09) | 0.084 |  | |  |  |
| PCT | 0.04(0.00-0.92) | 0.044 | 0.23(0.01-5.53) | 0.368 | 0.45(0.09-2.30) | 0.337 |  | |  |  |
| RAR | 1.11 (0.84 - 1.47) | 0.466 |  |  | 1.07(0.93-1.23) | 0.351 |  | |  |  |
| **All-cause mortality** | | | | |  |  |  | |  |  |
| Age | 1.04(1.02-1.07) | <0.001 | 1.04(1.02-1.07) | 0.002 | 1.03(1.02-1.04) | <0.001 | 1.03(1.01-1.04) | <0.001 | | |
| Sex(male) | 1.02(0.57-1.82) | 0.952 | 1.35(0.72-2.51) | 0.346 | 1.20(0.88-1.65) | 0.245 | 1.27(0.90-1.80) | | 0.178 |  |
| Smoking history | 0.58（0.33-1.04） | 0.068 |  |  | 0.74(0.56-1.00) | 0.047 | 0.76(0.56-1.02) | | 0.067 |  |
| Alcohol consumption history | 0.95(0.46-1.94) | 0.886 |  |  | 0.81(0.57-1.15) | 0.234 |  | |  |  |
| NYHA class Ⅲ-Ⅳ | 2.33(1.14-4.75) | 0.020 | 2.02(0.92-4.42) | 0.079 | 1.48(0.94-2.33) | 0.087 |  | |  |  |
| Coronary heart disease | 1.71(0.86-3.40) | 0.123 |  |  | 1.04(0.79-1.37) | 0.803 |  | |  |  |
| Dilated cardiomyopathy | 0.42(0.13-1.33) | 0.139 |  |  | 0.68(0.17-2.76) | 0.594 |  | |  |  |
| Valvular heart disease | 3.25(1.53-6.90) | 0.002 | 3.36(1.49-7.61) | 0.004 | 1.53(0.87-2.68) | 0.140 |  | |  |  |
| Atrial fibrillation | 2.11(1.21-3.68) | 0.008 | 1.32(0.73-2.39) | 0.358 | 1.25(0.89-1.76) | 0.195 |  | |  |  |
| Hypertension | 1.20(0.71-2.03) | 0.491 |  |  | 0.97(0.73-1.28) | 0.810 |  | |  |  |
| Diabetes | 1.08(0.63-1.87) | 0.780 |  |  | 1.24(0.91-1.68) | 0.176 |  | |  |  |
| ARNI/ACEI/ARB | 0.95(0.55-1.65) | 0.859 |  |  | 0.87(0.65-1.17) | 0.368 |  | |  |  |
| β-blockers | 0.52(0.31-0.89) | 0.016 | 0.58(0.33-0.99) | 0.047 | 0.94(0.71-1.24) | 0.671 |  | |  |  |
| Aldosterone receptor antagonists | 1.13(0.58-2.13) | 0.725 |  |  | 0.71(0.52-0.97) | 0.031 |  | |  |  |
| Uric acid | 1.001(0.999-1.002) | 0.402 |  |  | 1.000(1.000-1.001) | 0.499 |  | |  |  |
| Creatinine | 1.002 (1.000 - 1.005) | 0.043 | 1.002 (1.000 - 1.005) | 0.110 | 1.003(1.002-1.005) | <0.001 | 1.003(1.001-1.005) | | 0.002 |  |
| Potassium | 1.38(0.99-1.91) | 0.056 |  |  | 1.39(1.11-1.74) | 0.005 | 1.20(0.95-1.52) | | 0.123 |  |
| Sodium | 0.96(0.90-1.03) | 0.248 |  |  | 0.96(0.94-0.99) | 0.009 | 1.00(0.96-1.04) | | 0.957 |  |
| Chloride | 0.95(0.88-1.02) | 0.120 |  |  | 0.96(0.94-1.00) | 0.022 | 0.97(0.93-1.02) | | 0.203 |  |
| Hemoglobin | 0.99(0.98-1.01) | 0.218 |  |  | 0.99(0.99-1.00) | 0.046 | 1.00(0.99-1.01) | | 0.531 |  |
| PDW | 1.15(1.04-1.27) | 0.005 | 1.12(1.01-1.24) | 0.028 | 1.03(0.98-1.09) | 0.286 |  | |  |  |
| PCT | 0.14(0.00–8.00) | 0.342 |  |  | 0.21(0.03-1.68) | 0.141 |  | |  |  |
| RAR | 1.58(1.15-2.16) | 0.005 | 1.18(0.77-1.80) | 0.440 | 1.47(1.23-1.75) | <0.001 | 1.33(1.08-1.65) | | 0.007 |  |

**Abbreviations:** HFimpEF, heart failure with improved ejection fraction; HFrEF, heart failure with reduced ejection fraction; NYHA, New York Heart Association; ACEI, angiotensin converting enzyme inhibitor; ARB, angiotensin receptor blocker; ARNI, angiotensin receptor neprilysin inhibitor; PDW, platelet distribution width; PCT, plateletocrit; RAR, red blood cell distribution width/albumin ratio; HR, hazard ratio; CI, confidence interval.
